# Genomic characterization of intracranial teratomas using whole genome sequencing

**DOI:** 10.3389/fonc.2022.1013722

**Published:** 2022-11-15

**Authors:** Chao Zhang, Xiaoyu Zhou, Xiang Huang, Xinghua Ding, Yang Wang, Rong Zhang

**Affiliations:** ^1^ Department of Neurosurgery, Huashan Hospital, Shanghai Medical College, Fudan University, Shanghai, China; ^2^ Department of Pediatric Neurosurgery, Neurosurgical Institute of Fudan University, Shanghai, China; ^3^ Department of Pediatric Neurosurgery, Shanghai Clinical Medical Center of Neurosurgery, Shanghai, China; ^4^ Department of Pediatric Neurosurgery, National Center for Neurological Disorders, Shanghai, China; ^5^ Department of Neurosurgery, Putuo District People’s Hospital of Shanghai, Shanghai, China; ^6^ Department of Medicine, GenomiCare Biotechnology (Shanghai) Co. Ltd., Shanghai, China; ^7^ Department of Radiotherapy, Huashan Hospital, Fudan University, Shanghai, China

**Keywords:** teratoma, intracranial, genomic characterization, whole genome sequencing, pediatric

## Abstract

**Background:**

Intracranial teratoma is a rare neoplasm of the central nervous system, often classified into mature and immature types and occurs mainly in children and adolescents. To date, there has been no comprehensive genomic characterization analysis of teratoma due to its rarity of the cases.

**Methods:**

Forty-six patients with intracranial teratomas were collected and 22 of them underwent whole-exome sequencing, including 8 mature teratomas and 14 immature teratomas. A comprehensive analysis was performed to analyze somatic mutations, copy number variants (CNVs), mutational signatures, and Kyoto Encyclopedia of Genes and Genomes (KEGG) pathway in our cohort.

**Results:**

The most common somatic mutated gene in intracranial teratomas was CARD11 (18%) and IRS1 (18%), followed by PSMD11, RELN, RRAS2, SMC1A, SYNE1 and ZFHX3, with mutation rates of 14% for the latter six genes. Copy number variation was dominated by amplification, among which ARAF (50%), ATP2B3 (41%), GATA1 (41%), ATP6AP1 (36%), CCND2 (36%) and ZMYM3 (36%) were the most frequently amplified genes. Copy number deletion of *SETDB2* and *IL2* only appeared in immature teratoma (43% and 36%, respectively), but not in mature teratoma (*p* = 0.051 and 0.115, respectively). Prognostic analysis showed that TP53 mutations might be associated with poor prognosis of intracranial teratomas patients.

**Conclusions:**

Our study revealed the genetic characteristics of intracranial teratoma which might be valuable for guiding future targeted therapies.

## Introduction

Intracranial teratoma is a rare neoplasm of the central nervous system with unexplored molecular pathogenesis, accounting for approximately 0.4% of all primary intracranial tumors (1). Children are the most susceptible population, and intracranial teratoma accounts for a higher proportion of 2-4% of intracranial tumors in children (2, 3). The fifth edition of the 2016 WHO Classification of Tumors of the Central nervous system classifies teratoma into three categories: mature teratoma (MT), immature teratoma (IMT) and teratoma with somatic-type malignancy (4). Intracranial teratoma belongs to non-germinomatous germ cell tumors (NGGCT), but it is a subgroup with a better prognosis compared with other subgroups (including yolk sac tumors, choriocarcinomas, and embryonal carcinomas). The 5-year survival rate of mature teratoma is 87-100%, and that of immature teratoma is 33-71% (5–7).

Surgical resection is the main treatment for intracranial teratoma. The benefits of adjuvant therapy are controversial. Radiation therapy remains an important accepted treatment for intracranial teratoma (8). Mature teratoma is considered to have limited benefit from radiotherapy, but relapsed mature teratoma is sensitive to radiotherapy (9). However, no consensus has been reached on the dose and volume of radiotherapy (10, 11). Moreover, platinum-based chemotherapy has different efficacy for various subtypes (9). Therapeutic strategy aimed to improve quality of life and prolong progression-free and overall survival are ongoing.

Given their rarity, the literature on intracranial teratoma is limited, so the molecular profile of intracranial teratoma remains poorly understood. In the few molecular studies to date, intracranial teratoma has been included as part of intracranial germinoma or NGGCT, which may have overlooked its unique features (12–14). In this study, we collected 46 patients with intracranial teratomas and analyzed mutational landscape of 22 of them by whole-exome sequencing (WES), in order to identify new genetic targets which may provide new therapeutic strategies, and obtain biomarkers related to prognosis by combining molecular data and clinical features.

## Materials and methods

### Patients and sample collection

A total of 46 patients with intracranial teratoma were retrospectively enrolled from 2018 to 2020. The protocol has been approved by Huashan Hospital. Due to insufficient samples in some patients, subsequent experiments were performed only on tumor specimens and matched whole blood collected during resection surgery in 22 of these patients, and all patients provided informed consent. The pathological diagnosis was performed by experienced pathologists.

### Whole-exome sequencing

WES and analysis were performed at the Genomics Laboratory of GenomicCare Biotechnology (Shanghai, China), which is CLIA/CAP certified. DNA was extracted from serial thick sections (10 μm) cut from formaldehyde-fixed paraffin-embedded (FFPE) or fresh-frozen tumor blocks and matched peripheral blood leukocytes. The leukocytes were used as the germline DNA controls. The invasive tumor content was estimated by pathologists to ensure more than 20% of cells were tumor cells. For thawed tumor tissue or blood, DNA was extracted using the Maxwell RSC Blood DNA Kit (cat# AS1400, Promega, Madison, WI, USA) on a Maxwell RSC system (cat# AS4500, Promega). For FFPE sections, DNA was extracted using the MagMAX FFPE DNA/RNA Ultra Kit (cat# A31881, ThermoFisher, Waltham, MA, USA) on a KingFisher Flex system (ThermoFisher). The extracted DNA was sheared using a Covaris L220 sonicator, then the exome DNA was captured using the SureSelect Human All Exon V7 kit (cat# 5991-9039EN, Agilent, Santa Clara, CA, USA), prepared to libraries using the SureSelectXT Low Input Target Enrichment and Library Preparation system (cat# G9703-90000, Agilent, Santa Clara, CA USA), and sequenced on an Illumina NovaSeq-6000 sequencer (Illumina, San Diego, CA, USA) to generate 150×150 bp paired end reads. The mean depths were of 218 folds and 104 folds for the tumor and germline control DNA samples respectively. Image analysis and base calling was done using onboard RTA3 software (Illumina). After removing adapters and low-quality reads, the reads were aligned to NCBI human genome reference assembly hg19 using the Burrows‐Wheeler Aligner alignment algorithm and further processed using the Genome Analysis Toolkit (GATK, version 3.5), including the GATK Realigner Target Creator to identify regions that needed to be realigned.

### Somatic variant identification

After removing adapters and low-quality reads, the commercial Sentieon (version 201911) (15) running environment with default parameters was implemented to process the following steps sequentially: reads alignment to NCBI human genome reference assembly hg19 using the Burrows‐Wheeler Aligner (BWA) algorithm, duplication sorting, realignment and recalibration, and somatic mutation calling including single nucleotide variations (SNVs) and short insertion/deletions (INDELs). During the mutation calling stage, the reads from the tumor sample were compared with the paired blood from the same patient to generate the somatic mutation list. The called somatic mutations were then filtered, meaning to retain only the mutations with the variant allele frequency (VAF) >= 0.05 and supported by at least three reads, and annotated using the Variant Effect Predictor (VEP) package (16).

### Copy number variation

According to the haploid Copy Number (CN) calculation method published by Jarupon et al. (17), the ExomeCNV packageused to calculate CN at the exon level could estimate CN of a specific gene. A normalized depth-of-coverage ratio approach was used to identify CNV from the WES result of paired samples. Standard normal distribution was used to account for five sources of bias that would affect raw read counts, which include the size of exonic regions, batch effect, the quantity and quality of the sequencing data, local GC content, and genomic mappability. Only genes with more than 200 mapped reads in its tumor sample data or corresponding blood control sample data were kept. Genes with CN <= 1, 1< CN <=1.2, 3<= CN < 4, CN >=4 was defined as deletion, loss, gain, amplification, respectively and a minimum tumor content (purity) of 20% is required.

### Applying cancer-related gene filters

After calling SNV, CNV by the above steps, the resulting mutated genes were further filtered by intersecting with a group of cancer-related genes collected from two popular public cancer gene databases.

Part1 is from OncoKB curated cancer gene list (18). These genes are considered to be cancer genes by OncoKB, based on their inclusion in various different sequencing panels, the Sanger Cancer Gene Census, or Vogelstein et al. (2013). Part2 is from the ranked CIViC gene candidate table (19). This list is based on a survey of 90 commercially available clinical gene panels developed by 40 distinct institutes and companies.

The final list of cancer-related gene includes genes from Part1 and Part2 with a ‘panel_count’ value >= 2.

### Bioinformatic analysis

The mutational signature classification was based on COSMIC Mutational Signature (version 2 – March 2015), which was generated from studies performed by others (20–22). Tumor mutation burden (TMB) was defined as the total number of somatic nonsynonymous mutations in each sample according to a previous method for WES data (23). All autosomal microsatellite tracts containing 1–5 bp repeating subunits in length and comprising five or more repeats in GRCh37/hg19 were identified using MISA (http://pgrc.ipk-gatersleben.de/misa/misa.html) and used to calculate microsatellite instability score (MSI). MSI score was calculated by the number of unstable microsatellite sites/total valid sites. Homologous recombination deficiency (HRD) score was defined as the unweighted sum of loss of heterozygosity (LOH), telomeric allelic imbalance (TAI), and large-scale transition (LST) scores. The mutant-allele tumor heterogeneity (MATH) score was calculated by the width of the VAF distribution using maftools (24). Cosmic signature was calculated using SNV data by maftools, signature contribution is calculated by R package MutationalPatterns (https://github.com/UMCUGenetics/MutationalPatterns). Pathway map is modified on the basic output of an online tool PathwayMapper (http://www.pathwaymapper.org/). Kaplan-Meier plot of survival data is generated by the R package survminer. Other figures are generated by R package ggplot2 or maftools.

### Targetable alterations

The SNV, CNA and related clinical data is annotated by the OncoKB official python package oncokb-annotator (https://github.com/oncokb/oncokb-annotator). The information mentioned in the Results part is the output of script ClinicalDataAnnotator.py.

### Statistical analysis

SPSS Statistics 22.0 and R (https://cran.r-project.org) packages were employed in correlation analysis of clinical and biological variables with Pearson’s Chi-Square test or Fisher’s exact test for categorical variables and Mann-Whitney U test or Kruskal-Wallis H test for continuous variables as appropriate. The overall survival (OS) and progression-free survival (PFS) were calculated using the Kaplan-Meier method, and differences between variables were compared using log-rank test. *P* < 0.05 was considered statistically significant.

## Results

### Patient samples and clinical data

**Figure 1:**
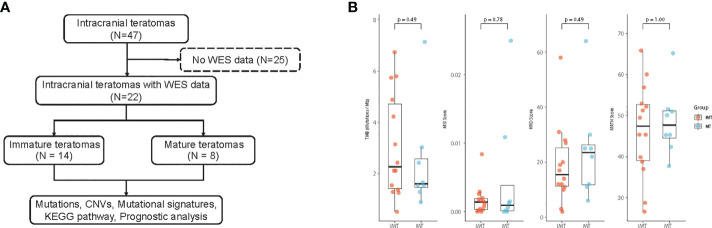
**(A)** Workflow of the study. **(B)** Comparison of Tumor mutational burden (TMB), Microsatellite instability (MSI), Homologous recombination deficiency (HRD) and the mutant-allele tumor heterogeneity (MATH) score between IMT and MT.

In total, 46 patients with intracranial teratomas were enrolled in the study. The detailed description of each patient is provided in **Table S1**. The median age of the patients was 14 years and 40 (87.0%) patients were male. According to histological classification, it can be divided into MT (25, 54.3%), IMT (5, 10.9%) and mixed teratoma (16, 34.8%). Since the prognosis of mixed teratoma is similar to that of IMT, mixed teratoma was also included in IMT group. That is, 25 (54.3%) of the 46 patients were in the MT group and 21 (45.7%) were in the IMT group (including pure IMT and mixed teratoma). We selected 22 patients for whole-exome sequencing, including 14 IMT and 8 MT (**Figure 1A**). Pineal region was the most common anatomic site of tumors, which occurs in 77.3% of patients. The clinical information of the 22 patients is summarized in **Table 1**. The clinicopathological characteristics of patients between IMT and MT groups were compared, and the results showed the proportion of patients receiving chemotherapy was higher in the IMT group than in the MT group (*p*=0.010). Other factors were not statistically different between the two groups (*p*>0.05).

**Table 1:** Clinicopathologic features of 22 intracranial teratomas patients with whole-exome sequencing.

Clinicopathological Characteristics	Mature teratoma (N = 8)	Immature teratoma (N = 14)	p value
	N (%)	N (%)	(Fisher’ exact test)
**Gender**			1
Female	1 (12.5)	1 (7.1)	
Male	7 (87.5)	13 (92.9)	
**Age, year**			1
<14	4 (50)	6 (42.9)	
≥14	4 (50)	8 (57.1)	
**Recurrence**			0.602
Yes	2 (25)	2 (14.3)	
No	6 (75)	12 (85.7)	
**Death**			0.273
Yes	0 (0)	3 (21.4)	
No	8 (100)	11 (78.6)	
**AFP**			0.183
Normal	6 (75)	5 (35.7)	
Elevated (>25ng/mL)	2 (25)	9 (64.3)	
**β-HCG**			0.515
Normal	8 (100)	12 (85.7)	
Elevated (>50IU/L)	0 (0)	2 (14.3)	
**Radiotherapy**			1
Yes	4 (50)	6 (42.9)	
No	4 (50)	8 (57.1)	
**Chemotherapy**			0.010
Yes	4 (50)	14 (100)	
No	4 (50)	0 (0)	
**Gamma knife**			1
Yes	0 (0)	1 (7.1)	
No	8 (100)	13 (92.9)	

### Molecular characterization in intracranial teratomas genomes

Whole exome sequencing (WES) was performed on tumor and matched whole blood samples of 22 intracranial teratoma patients. After mutation calling and tumor-related gene list filtering, 2193 somatic mutations, including 1945 SNVs and 248 indels (insertions and deletions) were detected, which gave the median somatic mutation per patient at 74 (**Table S2**). The median TMB was 3.69 mutation/MB in the IMT group and 1.6 mutation/MB in the MT group, but there was no statistical difference between the two groups. (*p*=0.49) (**Figure 1B**). MSI, HRD and MATH score also did not differ between the two groups (*p*=0.78, 0.49 and 1.00, respectively).

**Figure 2:**
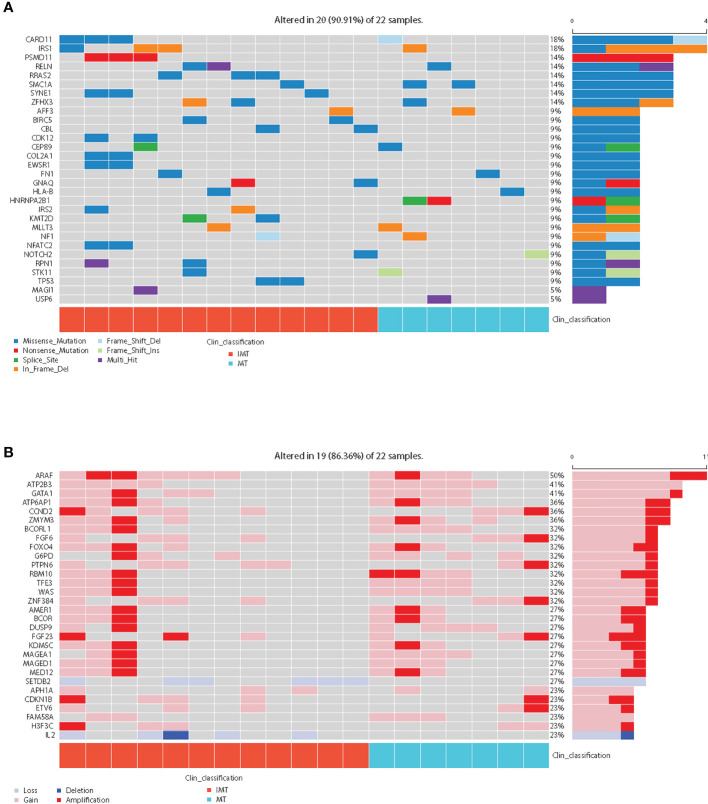
Mutational landscape of the 22 intracranial teratoma patients. **(A)** The somatic mutation status of cancer-related genes. The percentage and horizontal bar on the right of each row indicate the fraction of patients with mutations in the corresponding genes and the composition of the types of mutations as color coded below the plot. In clinical classification, red represents IMT and blue represents MT. **(B)** The top 30 prevalence genes in the cohort with CNVs (amplification, gain, loss, and deletion were defined as copy number (CN)≥4, 3≤CN<4, 1<CN ≤ 1.2, and CN ≤ 1, respectively). In clinical classification, red represents IMT and blue represents MT.

Among cancer-related genes (**Table S3**), *CARD11*(18%) and *IRS1*(18%) were the most common somatic mutated genes, followed by *PSMD11*, *RELN*, *RRAS2*, *SMC1A*, *SYNE1* and *ZFHX3*, and the mutation rates of the latter six genes were all 14%. (**Figure 2A**). However, the mutation frequencies of cancer-related genes in IMT and MT were not statistically different (**Table S4**). Recurrent copy number amplifications were observed in ARAF (50%), ATP2B3 (41%), GATA1 (41%), ATP6AP1 (36%), CCND2 (36%), and ZMYM3 (36%) (**Figure 2B**; **Table S5**). It is worth noting that copy number variations (CNVs) of SETDB2 and IL2 only occur in IMT (42.9% and 35.7%, respectively), but the difference was not statistically significant (p=0.051 and 0.115, respectively) (**Table S6**).

**Figure 3:**
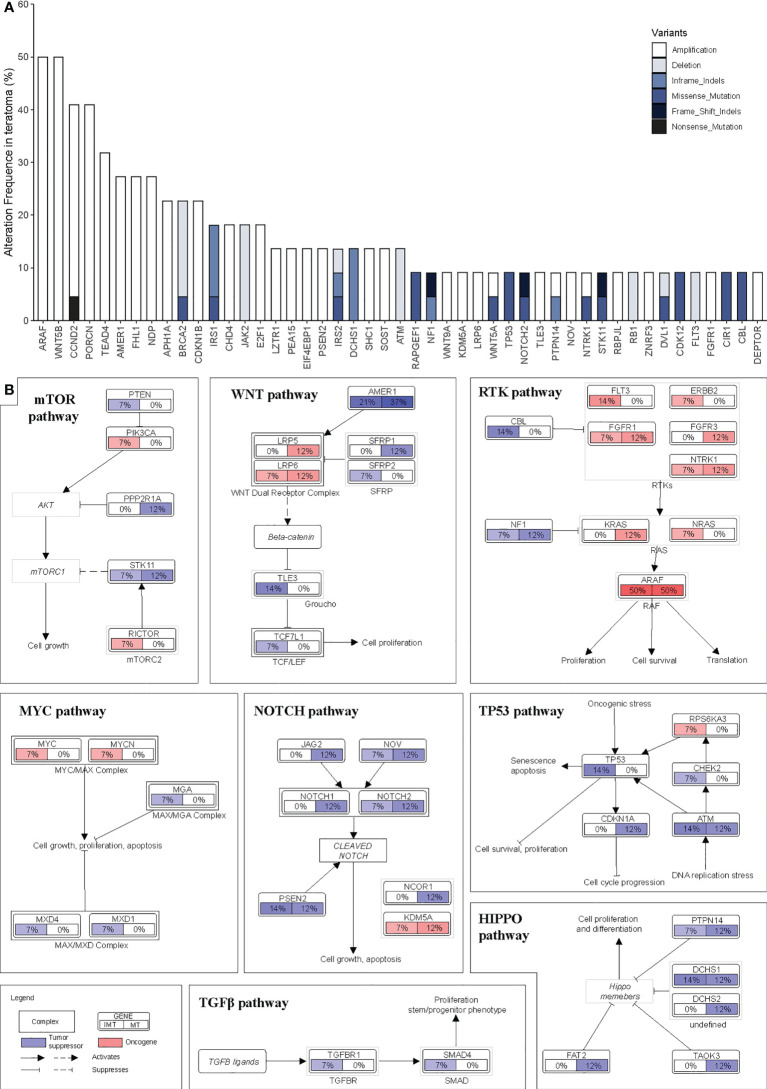
Landscape of structural genomic alterations in signaling pathway. **(A)** Frequency of recurrent mutations and copy number alterations in intracranial teratomas ranked by their prevalence. **(B)** Pathway diagrams showing the percentage of samples from each intracranial teratoma subtype with structural genomic alterations in major genes from HIPPO, TP53, MYC, TGF-β, NOTCH, mTOR, RTK-RAS and WNT pathways. Red and blue mean oncogene and tumor suppressor, respectively.

We integrated the mutation and CNVs profiles of the genes, as shown in **Figure 3A**. Overall, there were more CNV events than somatic mutation events. These genes converged into 7 main oncogenic signaling pathways: RTK-RAS (altered in 95.5% of tumors), WNT (86.4%), NOTCH (72.7%), HIPPO (54.5%), TP53 (18.2%), MYC (13.6%) and TGF-β (9.1%) (**Table S7** and **S8**). The alterations of major genes in these pathways in different subgroups are shown in **Figure 3B**. In every pathway, the proportion of alterations in genes varies by subgroup. However, the rates of alterations in all the pathways did not differ significantly between the two groups (*p*>0.05) (**Table S7**).

Next, the germline mutant genes were analyzed. Only two IMT patients each had one pathogenic germline mutation, *MUTYH* and *TP53*. The low mutation frequency made it impossible to analyze the difference between IMT and MT.

### Mutational spectrum and mutational signatures

**Figure 4:**
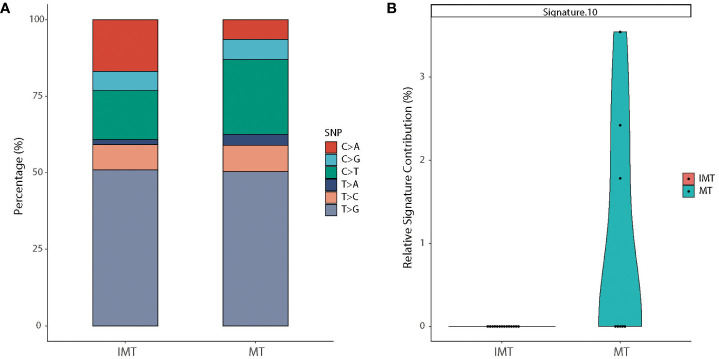
Mutational spectrum and mutational signatures in IMT and MT. **(A)** Stacked bar graph of the percentages of six single-nucleotide substitutions in IMT (left) and MT (right) groups. **(B)** Relative contribution of signature 10 which was statistically different between the two subtypes.

T>G was the most common substitution in intracranial teratoma patients (50.9% in IMT and 50.4% in MT), followed by C>T (16.0% in IMT and 24.4% in MT) and C>A (16.8% in IMT and 6.4% in MT) (**Figure 4A**). The proportion of 6 base substitutions did not differ between IMT and MT (*p*>0.05). In order to determine the relationship between mutation frequency distribution of tumor samples and cosmic signature, we calculated the contributions of individual mutational signatures and identified main signatures within the tumors tested, including signature 3 (associated with germline and somatic BRCA1/2 mutations), signatures 1 (associated with age), and signature 16 (unknown etiology) (**Figure S1**). Among the 30 signatures, signature 10 (associated with POLE mutations) showed higher relative contributions in MT than in IMT (*p*=0.019) (**Figure 4B**).

### Potentially actionable targets in intracranial teratomas

We assessed the potentially actionability of genomic alteration detected by WES using OncoKB precision oncology knowledge database (https://www.oncokb.org/) (25) and found that only 4 patients (18.2%) had targetable alterations, all of which were oncoKB level 4. They were *ARID1A* (tazemetostat, PLX2853), *NF1* (trametinib, cobimetinib) and *PTEN* (GSK2636771, AZD8186) in three IMT patients, and *KRAS* (trametinib, cobimetinib, binimetinib) in one MT patient (**Table S9**).

### Survival analysis of intracranial teratoma patients

**Figure 5:**
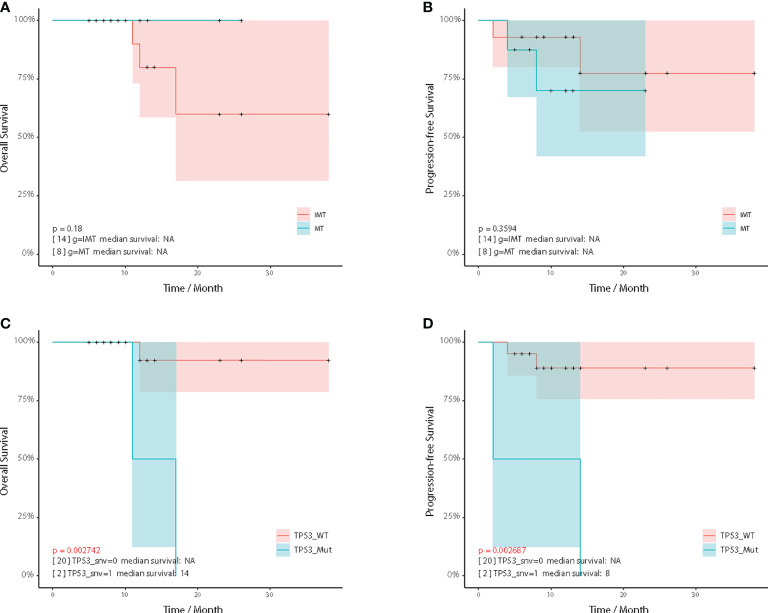
Kaplan–Meier analysis. Comparison of overall survival **(A)** and progression-free survival **(B)** between the IMT and MT subgroup. Comparison of overall survival **(C)** and progression-free survival **(D)** between the TP53 wildtype and TP53 mutated patients.

To identify prognostic factors of intracranial teratomas, we performed survival analysis of essential clinical factors and genetic biomarkers (**Tables S10**, **S11**). Four patients developed disease relapse (two of them died), and one patient died without relapse (**Table S1**). Compared with the IMT group, the overall survival time (OS) of the MT group tended to be prolonged, but there was no statistical difference (*p*=0.180) (**Figure 5A**). Progression-free survival (PFS) did not differ between the two groups (*p*=0.359) (**Figure 5B**). For genetic biomarkers we analyzed by univariate Kaplan-Meier method, the mutations of 3 genes (*CD3EAP*, *TP53* and *PCDH17*) and CNVs of *TIMP3* were associated with OS. The mutations of 6 genes (*EFNB3*, *KRT7*, *NF1*, *HNRNPA2B1* and *TP53*) and CNVs of 2 genes (*HIST2H3D* and *ERG*) were associated with PFS. We noticed that patients with *TP53* mutations had shorter OS and PFS than those without *TP53* mutation (**Figures 5C, D**). For the clinical factors, including gender, age, AFP, β-HCG, radiotherapy, chemotherapy and gamma knife, no statistically significant correlation with OS and PFS was found.

## Discussion

In this study, we comprehensively characterized genomic profiles of an intracranial teratoma cohort, and analyzed the potential clinical correlation and implications of these molecular signatures. We have systematically analyzed copy number variations, somatic mutations, germline mutations, mutation spectrum and mutational signatures by WES, in order to establish a more widespread genomic landscape and identified molecular differences among different subtypes.

*KIT* was the most significantly mutated gene in previous studies (14, 26). *KIT* is a ligand-dependent kinase, and its mutation will constitutively activate the downstream effectors of KIT/RAS pathway (27, 28). *KIT* has a high mutation frequency in germinoma, but it was rare in NGGCT. In our study, *CARD11*(caspase recruitment domain family member 11) (mutated in 18% intracranial teratomas), *IRS1*(insulin receptor substrate 1) (18%), *PSMD11*(16%), *RELN*(16%), *RRAS2*(16%), *SMC1A*(16%), *SYNE1*(16%) and *ZFHX3*(16%) were the tumor-related genes with the highest mutation rates in our cohort. Among the CNVs, ARAF (50%), ATP2B3 (41%), GATA1 (41%), ATP6AP1 (36%), CCND2 (36%), and ZMYM3 (36%) were the most frequently amplified genes. *CARD11* encodes a protein belonging to the membrane-associated guanylate kinases (MAGUK) family. Mutations in *CARD11* are associated with apoptosis and abnormal activation of NF-KappaB, which may lead to compromised adaptive immunity, leading to various immunological diseases in patients (29). High expression of CARD11 is associated with shorter overall survival in uveal melanoma (30). *IRS1* encodes a protein that is phosphorylated by the insulin receptor tyrosine kinase. Activation of *IRS* is transmitted to insulin/IGF1 signaling, which plays an important role in brain activity, and IRS1 is required for spinal maturation and neurogenesis (31). *ARAF* is a highly conserved serine/threonine kinase that controls ERBB3 expression in lung cancer, thereby inhibiting *AKT* activation and subsequently inhibiting tumor metastasis (32). But the role of these genes in central nervous system tumors remains unknown and need further investigation.

The median TMB for intracranial teratomas was 2.1 mutations/Mb, which is a relatively low mutation burden among various human cancer types (33). It is much smaller than that in non-small cell lung cancer and melanoma, which have a higher proportion of people benefiting from immunotherapy (34, 35). The high phenotype of MSI, another biomarker of immunotherapy, shows that patients have the highest probability of responding to PD-1 inhibitors (36). However, there were no patients with MSI-high (MSI score>0.035) in our cohort. All these results suggest that immunotherapy may have limited benefit in intracranial teratoma. Although there are still limited studies on intracranial teratoma, immunotherapy has little effect on central nervous system tumors due to its unique immune microenvironment and the existence of blood-brain barrier (37). Glioblastoma is a typical tumor of high intrinsic resistance and high adaptive resistance, and Checkmate 143 clinical trial shows that less than 10% of patients responded to immunotherapy (38). Glioblastoma is not an irreversibly ‘cold’ tumor, several therapies targeting myeloid-mediated immunosuppression and dysfunctional antigen presentation are in development (39). With the increased understanding of the tumor microenvironment and the development of clinical trials, it is hoped that the benefit of immunotherapy in patients with central nervous system tumors, including intracranial teratoma, will be improved in the future.

Our study found 7 main oncogenic signaling pathways were involved in intracranial teratoma. The proportion of patients with gene variations (including mutations and CNVs) in RTK-RAS, WNT, NOTCH, TP53 and mTOR pathway was relatively high. Among them, RTK-RAS had the highest proportion, and 21 of the 22 patients had gene variations, including 15 patients with 32 mutations and 15 patients with 32 CNVs (9 patients with both mutations and CNVs). All these findings provide promising therapeutic targets for clinical practice and contribute to the development of new therapeutic strategies. For example, overexpression of mTOR signaling pathway imply that mTOR inhibitor may be a suitable option for these patients.

There were only 4 patients had targetable alterations of level 4 by OncoKB database. Level 4 means alterations associated with compelling biologic evidence of predictive value but neither biomarker nor drug are standard of care (25). Although the level of evidence for targeted therapy is not high, the promise of whole-exome sequencing to identify targeted therapies provides more strategies to improve the outcomes of patients with intracranial teratoma.

We also identified several genes whose CNVs frequencies differed between MT and IMT, although the differences were not statistically significant due to the small number of patients. CNVs of SETDB2 and IL2 occurred only in IMT. SETDB2 is a histone H3 lysine 9 (H3K9) trimethyltransferase, and its overexpression is associated with poor prognosis of gastric cancer patients (40). IL2 is an immune-stimulating cytokine of key immune cells and plays an important role in antigen-stimulated immune responses (41). These can provide information about the molecular differences between the two subgroups.

The prognosis of patients with intracranial teratoma is good, especially mature teratoma. Of the 22 patients, 4 (including 2 MT and 2 IMT) relapsed and 3 (all IMT) died. Two of the IMT cases with both recurrence and death included a malignant component in histology, and patients with a malignant component tend to have a poor prognosis. Both patients had TP53 mutations, and survival analysis showed that patients with TP53 mutations had shorter OS and PFS, suggesting that TP53 may be associated with histological malignant components and poor prognosis. There are few related studies, and a case report of TP53-mutated intracranial immature teratoma has been reported (42). However, the remaining two patients with recurrence were mature teratoma. We tried to analyze the shared mutations and CNVs of these two patients, but there was no statistical difference in survival analysis because there were only two patients. We have attached the sequencing data and clinical information in the supplementary tables, and we look forward to a larger cohort of intracranial teratomas in the future that can be combined with our cohort to provide patients with more effective biomarkers.

In summary, we present a genetic atlas of the intracranial teratoma population, and found disparities in the mutation landscapes of different subtypes, which deepen our understanding of the disease. CARD11 and IRS1 were the genes with the highest mutation rates in our cohort. Among the CNVs, ARAF, ATP2B3 and GATA1 were the most frequently amplified genes. Our study identified frequent somatic alterations and copy number variants in RTK-Ras, WNT, NOTCH, TP53 and mTOR signaling pathways. These pathway findings provide potential therapeutic targets for novel therapeutic strategies. We also identified TP53 mutations may be associated with shorter OS and PFS. Our findings could be informative to establish more effective treatment and diagnostic strategies for intracranial teratoma patients based on the molecular genetic information of their tumors.

## Data availability statement

Data used by graphs is available at supplemental tables. Original VCF files are available at Chinese National Genomics Data Center (https://ngdc.cncb.ac.cn), project accession number PRJCA012808.

## Ethics statement

The protocol has been approved by Huashan Hospital. The patients/participants provided their written informed consent to participate in this study.

## Author contributions

CZ and XZ researched and drafted the article. XH and XD collected clinical data and tumor specimens. YW and RZ supervised the content. All authors wrote, reviewed, and edited the manuscript before submission. All authors contributed to the article and approved the submitted version.

## Funding

This work was supported by grants from the Health System Independent Innovation Science Foundation of Shanghai Putuo District (Ptkwws201814).

## Conflict of interest

Author XZ was employed by the company GenomiCare Biotechnology (Shanghai) Co. Ltd., Shanghai, China. The remaining authors declare that the research was conducted in the absence of any commercial or financial relationships that could be construed as a potential conflict of interest.

## Publisher’s note

All claims expressed in this article are solely those of the authors and do not necessarily represent those of their affiliated organizations, or those of the publisher, the editors and the reviewers. Any product that may be evaluated in this article, or claim that may be made by its manufacturer, is not guaranteed or endorsed by the publisher.
