# Supplementary figures and images for: Genomic characterization of intracranial teratomas using whole genome sequencing
Source: Front Oncol. 2022 Nov 15;12:1013722. doi: 10.3389/fonc.2022.1013722 (PMC9706722; doi:10.3389/fonc.2022.1013722)

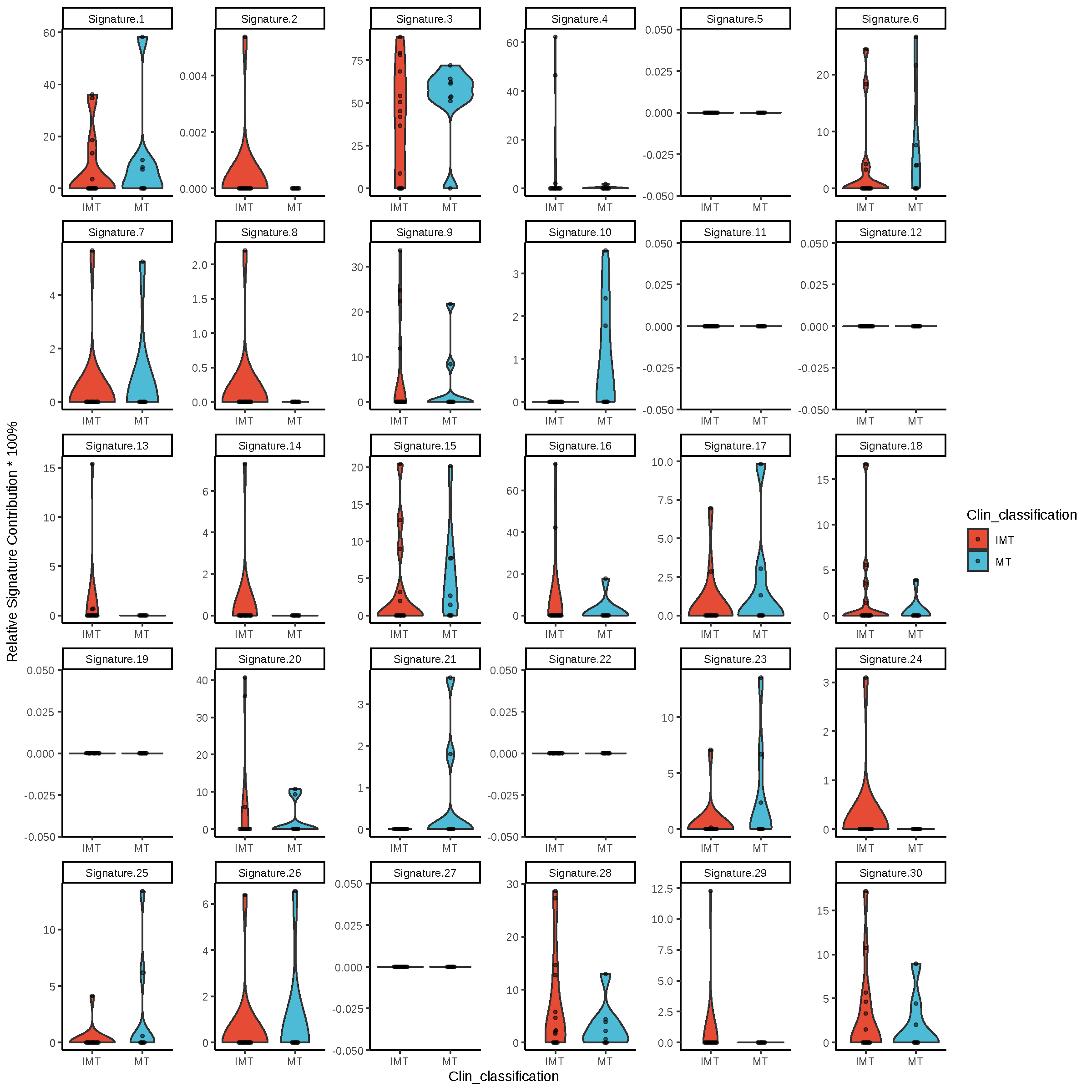

Supplement: Supplementary file 2 [file Image_1.tiff]
